# Disrupting Cell Cycle Machinery: CREPT Is an Emerging Target in Cancer Therapy

**DOI:** 10.3390/cancers17142401

**Published:** 2025-07-19

**Authors:** Umar Farooq, Jun Li, Zhijie Chang

**Affiliations:** 1 Jinfeng Laboratory, No. 313 Jinyue Road, High-Tech Zone, Chongqing 401329, China; umarfarooq@jflab.ac.cn; 2 State Key Laboratory of Membrane Biology, School of Basic Medical Sciences, School of Medicine, Tsinghua University, Beijing 100084, China

**Keywords:** CREPT, cyclin D1, cell cycle, cancer, therapeutic target

## Abstract

The transcriptional co-factor cell-cycle-related and expression-elevated protein in tumors (CREPT) has emerged as a critical driver of the cell cycle and a significant contributor to tumorigenesis. The aberrant expression or upregulation of CREPT boosts multiple signaling pathways, including Wnt/β-catenin, STAT3 and NF-κB/TNFR2, which are frequently dysregulated in various cancers and are associated with poor overall survival. In preclinical studies, CREPT knockdown via shRNA has demonstrated sustained tumor growth regression. Recent researches have uncovered additional functions of CREPT, including roles in metabolic regulation, tissue repair, and microenvironmental remodeling, further establishing it as a pleiotropic transcriptional regulator. Currently, there is no therapeutic agent that directly inhibits CREPT expression in clinic. However, miRNAs and other methods have been used to target CREPT, which have yielded useful results in inhibiting tumor growth. In this review, we discuss the role of CREPT in the hallmarks of cancer and propose that targeting CREPT will reverse tumor growth and may improve the immune checkpoint inhibitors in combination in CREPT-driven cancers.

## Simple Summary

Cancer is a leading cause of death worldwide, and researchers are continuously working to uncover its drivers and new therapeutic targets. In this review, we explain the role of the oncogenic protein CREPT that is overexpressed in the vast majority of cancers, leading to poor overall survival. CREPT ablation has shown sustained tumor regression in vitro and in mouse models. CREPT activation mediates cancer progression by enhancing the cell cycle by promoting chromatin loop formation. Thus, selectively targeting CREPT expression may provide alternative strategies for inhibiting abnormal cell division, metastasis, and tumor growth, and may improve the efficacy of the currently available therapies in combination, and will be key to reversing cancerous growth in patients with CREPT-driven cancers.

## 1. Introduction

Tumor development is initiated by perturbation of the cell cycle, resulting in aberrant and uncontrolled cell growth, involving the abnormal expression of both oncogenes and tumor suppressor genes [1]. Oncogenes typically promote cell growth, and mutations or epigenetic alterations can become overactive, leading to uncontrolled cell growth and division [2]. In contrast, tumor suppressor genes are typically responsible for preventing uncontrolled cell division. These genes are often inactivated or lose their function in cancer cells [3]. This imbalance between oncogenes and tumor suppressor genes allows cells to grow uncontrollably, resulting in tumor initiation and progression [4]. Disruption of these genes influences the activity or configuration of their encoded proteins, which is often observed in cancer. Activated oncogenes facilitate cellular proliferation and are important for cancer development [5].

The oncogenic gene cell-cycle-related and expression-elevated protein in tumors (CREPT), sometimes referred to as the regulation of the nuclear pre-mRNA domain containing 1 B (RPRD1B), was recognized as an oncoprotein two decades ago and was found to enhance transcription by promoting chromatin loop formation. In humans, CREPT (gene code 58490) is located on chromosome 20, which comprises five exons that encode a protein sequence of 326 amino acids [6]. CREPT plays a critical role in tumorigenesis, and its elevated expression is associated with cell proliferation and poor outcomes in various cancers (Table 1) [7,8,9,10,11,12,13]. The main function of CREPT is to contribute through transcriptional activation, which accelerates cell cycle progression and facilitates tumor growth [6,14]. This biological effect is primarily underscored by its critical role in the Wnt/β-catenin, STAT3 and NF-kB signaling pathways. These pathways are essential and govern important cellular processes such as cell division, differentiation, proliferation, and apoptosis [14]. Dysregulation of these pathways is a hallmark of neoplastic transformation in various cancers [15]. CREPT promotes β-catenin transcriptional activity by maintaining its interaction with the transcriptional co-activator and activating the activity of enhancers in a p300-dependent manner, increasing Wnt target gene expression, and facilitating tumor growth and development. This ability contributes to the alteration of oncogenic pathways, making CREPT a major driver of cancer progression in breast cancer, glioma, and hepatocellular carcinoma [7,16,17]. Furthermore, CREPT plays a critical role in altering the key genes involved in cell adhesion, angiogenesis, inflammation, and extracellular matrix remodeling, and is an essential player in driving triple-negative breast cancer [18].

CREPT plays a critical role in chromatin remodeling, RNA processing, and DNA repair by regulating the Wnt/β-catenin signaling pathway. For example, Liu et al. investigated its role in chromatin modification and demonstrated that CREPT interacts with histone deacetylase 2 (HDAC2), constructing a regulatory loop that refines the Wnt signaling pathway [19]. Another study showed that CREPT disrupts histone deacetylase 1 (HDAC1) function in cancer and promotes the expression of other oncogenes [20]. Furthermore, CREPT plays an essential role in the regulation of the cell cycle at various stages. For instance, CREPT upregulation enhances cyclin D1 expression and promotes the G1/S phase transition [21]. Another study showed that CREPT interacts with Aurora B kinase, which accelerates the upregulation of Cyclin B1 and increases the G2/M transition, contributing to cancer cell proliferation and tumor growth [22]. In addition to its role in oncogenesis, the role of CREPT in response to cellular stress and inflammation has gained increased recognition, broadening its significance to other diseases, including neurodegeneration and fibrosis, tissue repair, and metabolism [23,24,25].

CREPT is clinically significant as a predictive biomarker for cancer diagnosis and a therapeutic target [26]. High CREPT expression occurs in approximately 47 to 78% of tumors compared to normal tissues, which has been confirmed in various cancers through immunohistochemistry and Western blot analysis in samples obtained from various cancer patients [11,17,27,28]. In renal cell carcinoma, CREPT expression was elevated in tumor tissues compared to adjacent normal tissues, with high nuclear localization confirmed by IHC using a large cohort of (*n* = 90). Increased CREPT levels correlated significantly with reduced overall and disease-free survival (*p* < 0.05, * *p* < 0.01) [29]. Similar associations have been reported in gastric, colorectal, hepatocellular, and breast cancers, where CREPT expression is often upregulated and linked to poor prognosis based on Kaplan–Meier analyses [18,30,31]. In retroperitoneal leiomyosarcoma, the overall survival with CREPT expression was 33 months, while without CREPT expression was estimated as 60 months [32]. Similarly, in esophageal squamous cell carcinoma, the five-year overall survival estimated for CREPT was as high as 40.9%, while without CREPT, it was as high as 50.1% [26].

The elevated expression of CREPT promotes cancer progression and contributes to poor prognosis. Research has shown that CREPT knockdown using Adeno-associated viruses (AAV) delivery and targeting via microRNAs, including mir-449-5P, microRNA-501–3p, microRNA-449b-5p, and microRNA-383, has suppressed its expression and interrupted the aberrant Wnt signaling, which leads to the suppression of tumor growth [7,11,17,18,27]. Therefore, there is need for preclinical and clinical approaches to evaluate how targeting CREPT in combination with other targeting therapy including immune checkpoint inhibitors could improve the prognosis.

This review explores the multifaceted role of CREPT in cancer, with a focus on its mechanistic role in key signaling pathways, such as its interaction with cell cycle regulators, Wnt/β-catenin, and the tumor necrosis factor (TNF) signaling pathway. Owing to its involvement in these key signaling pathways, more attention is needed to uncover how elevated CREPT expression drives cancer cells to influence their microenvironment, which could have implications in cancer immunity.

## 2. Interaction of CREPT with RNA Polymerase II

**Figure 1:**
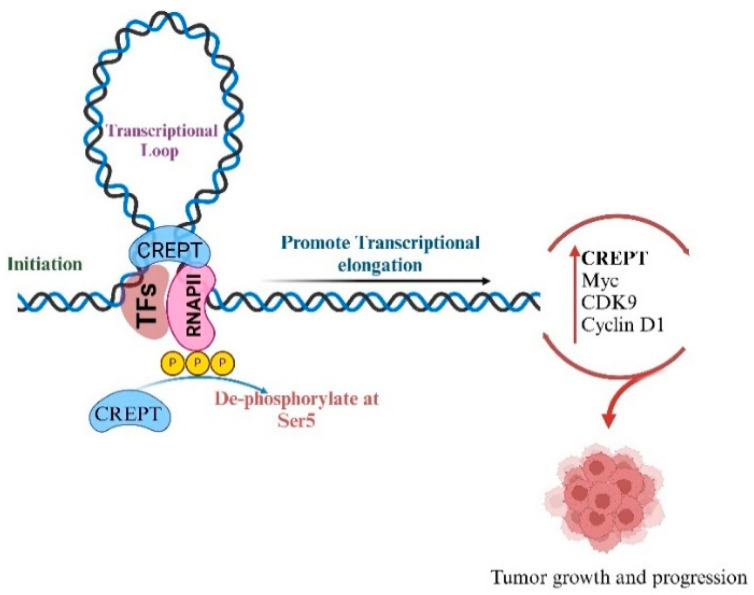
CREPT interacts with RNAPII at the promoter region of cyclin D1 and creates a loop that promotes the transcription from initiation to elongation. CREPT also maintains the phosphorylated status of RNAPII at the promoter and increases its transcriptional activity of other key genes like MYC and CDK9 and increases tumor growth and cell proliferation.

CREPT interacts with RNA polymerase II (RNAPII) to facilitate and accelerate chromatin loop formation, thereby promoting transcription [18]. The N-terminal domain of CREPT binds to the C-terminal domain of RNAPII (CTD-RNAPII). The terminal residues of the CREPT CTD-interacting domain (CID) amino acids Gln 281 and Arg285 are responsible for interaction and stabilization with RNAPII. Their interaction facilitates the transcription of other factors, such as serine/threonine cyclin-dependent kinase 9 (CDK9), and promotes the transition from initiation to elongation [33]. This is critical for full-length transcript synthesis and the recruitment of other key genes, such as cyclin D1, which is crucial for the G1/S phase transition and leads to cell cycle progression and proliferation [14]. Overexpression of cyclin D1 is common in many cancers, where it can lead to uncontrolled cell cycle progression by passing the normal checkpoints that prevent the proliferation of damaged or mutated cells. This can accelerate tumor growth and metastasis [34,35]. Additionally, the upregulation of cyclin D1 can be driven by mutations in genes that regulate its synthesis, such as oncogenes, or through altered signaling pathways. In cancers, failure to properly regulate the G1/S transition due to persistent overexpression of cyclin D1 and other cell cycle regulators contributes to the loss of cell cycle control, enabling tumor cells to proliferate uncontrollably [36,37]. Targeting these dysregulated cell cycle pathways holds promise for cancer therapies, such as restoring normal cell cycle checkpoints or inhibiting key regulatory proteins such as cyclin D1, which could potentially halt tumor growth and reduce cancer progression [38,39]. The overexpression of cyclin D1 in cancer is mediated by CREPT interaction with the C-terminal domain (CTD) of RNAPII, which modulates the phosphorylation status of the CTD and translocates with RNAP II from promoter regions to 3′-untranslated regions (3′UTR) during in vivo transcription. Primarily mediated by the phosphorylation status of different CTD residues, such as CTD S5 and S7, RNAPII is phosphorylated in the target gene promoter region (Figure 1) [33]. This enables RNAPII to engage in specific transcriptional phases, including initiation, elongation, and termination. Thus, the ability of CREPT to increase phosphorylation also contributes to the enhanced transcription of other critical oncogenes, including CDK9 and MYC [9]. At the molecular level, the Arg 285 residue of the CREPT CID domain interacts with the PEST domain of MYC and serves as a critical mediator and initiates gene transcription. This study also found that CREPT plays an important role in mediating the crosstalk between the acetylation and phosphorylation status of RNAPII, whereas CREPT knockdown impairs deacetylase and phosphatase activity [40]. Furthermore, CREPT also recruits RNAPII to the transcriptional region of MYC, which plays a crucial role in the smooth and coordinated transcriptional process that accelerates tumor growth and development and offers a therapeutic strategy targeting their complex in cancer treatment [9]. It targets CREPT and RNAPII activity and the transcription of MYC, cyclin D1, and other oncogenes that are involved in cancer progression.

## 3. CREPT Untranslated Regions and Regulation Through MicroRNAs

**Figure 2:**
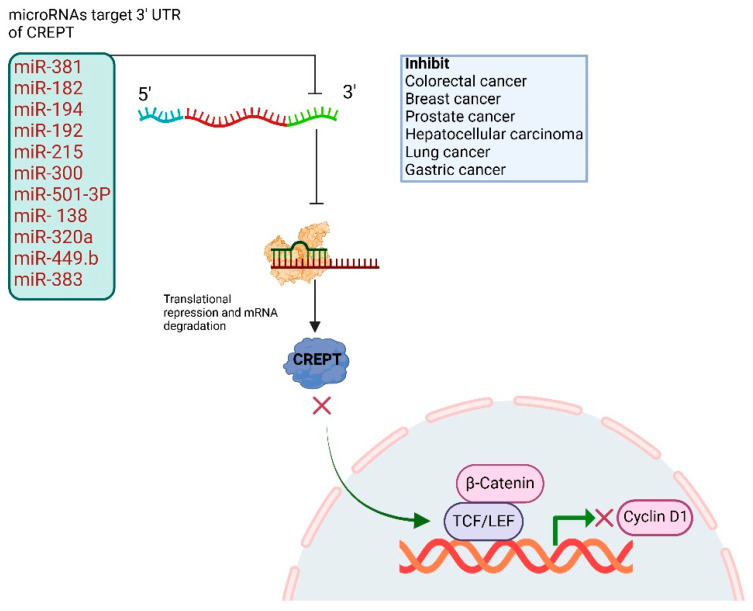
Schematic representation of CREPT 3′ UTR, which can be targeted through several miRNAs in cancer and has shown inhibitory effects in tumor growth, cell division, and proliferation.

MicroRNAs (miRNAs), which play an important role in post-transcriptional regulation, have been extensively studied. These are small non-coding RNAs found in bodily fluids and are approximately 20–22 nucleotides that generally cause a reduction in protein synthesis through translational repression [41,42]. miRNAs can target both 3′- and 5-untranslated regions as well as coding regions, but their main target is often the 3′- untranslated region (3′-UTR) [43,44]. The 3′-UTR of CREPT consists of 2486 nucleotides and serves as a key regulatory element [33]. Several miRNAs have been identified as regulators of CREPT, interacting with its 3′-UTR and altering gene expression (Figure 2) [11,17,45]. Jian et al. used prediction tools, such as miRanda and TargetScan, and identified multiple miRNAs with conserved binding sites in the CREPT 3′UTR, including miR-381, miR-182, miR-194, miR-192, miR-215, and miR-300 [17]. In glioma cells, CREPT expression is inversely correlated with miR-596 expression. This study further demonstrated that CREPT knockdown or overexpression of miR-596 suppressed cell proliferation and migration through the Wnt/β-catenin signaling pathway [45]. Among these miRNAs, miR-383 was found to directly regulate CREPT through its 3′-UTR in colorectal cancer, exhibiting an opposite correlation. The direct interaction between miR-383 and the 3′-UTR was confirmed using a luciferase assay [45]. Additionally, another study revealed that miR-501-3p targets the CREPT 3′-UTR and downregulates its expression level, resulting in cell cycle arrest mediated by downregulating cyclin D1 expression. This inhibitory effect of miR-501-3p was alleviated by CREPT overexpression in prostate cancer [46]. Liang et al. have revealed the regulatory roles of miR-138 and CREPT in breast cancer cells and tissues. It was discovered that miR-138 is a negative regulator of CREPT by targeting its 3′-UTR and suppressing its expression, leading to the inhibition of breast cancer cell proliferation and tumor progression. This study further revealed that the downregulation of miR-138 correlates with CREPT overexpression in breast cancer. Luciferase reporter assays showed that restoring miR-138 expression reduced CREPT expression levels, while upon CREPT 3′-UTR mutation, the interaction between both was disrupted, confirming the specificity of the miR-138’s/CREPT axis in breast cancer cell progression [11]. These studies demonstrate that the 3′-UTR of CREPT plays a critical role in its regulation in cancer and could be targeted by miRNAs. Further research is required to uncover the molecular mechanisms by which CREPT UTRs can be targeted, focusing on their influence on post-transcriptional gene expression and alternative splicing. Furthermore, suppressing CREPT expression may sensitize tumors to immunotherapeutic approaches and therefore requires combinatorial studies by using antisense oligonucleotide and small molecule inhibitors.

## 4. CREPT’s Role in Cell Cycle: G1/S and G2/M Transition

**Figure 3:**
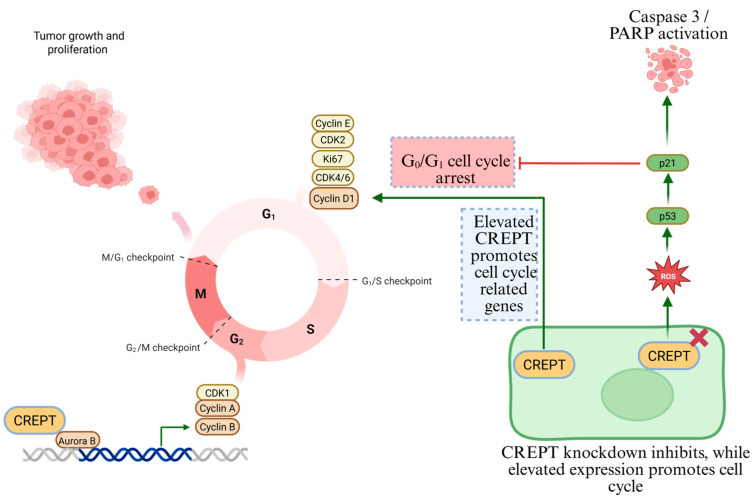
Model of CREPT regulating cell cycle progression in cancer. CREPT plays a critical role in both G1/S and G2/M transitions mainly by upregulating cyclin D1 and cyclin B expression, respectively.

The cell division cycle is composed of four phases: G1, DNA synthesis (S), G2, and mitosis (M), which are essential for multicellular growth and division [47]. Cellular events such as DNA replication, transcription, and translation occur in a cell-cycle-dependent manner, which is primarily initiated by the cell upon external stimuli and regulated by various checkpoints [47]. This process is primarily regulated by cyclin-dependent kinases (CDKs), which are stimulated by multiple growth factors [48]. The G1 phase is a critical period and the most extensively studied phase of the cell cycle, in which the cell expands in size and prepares for DNA replication by RNA synthesis and the generation of essential proteins. This phase is tightly regulated by CDKs and tumor suppressor proteins to ensure proper cell cycle progression. Perturbation at this phase enhances reliance on other cell cycle regulatory mechanisms, which remain unclear [48]. DNA replication errors or any other malfunction during cell division can be delayed at checkpoints and exit the cell cycle. However, cancer-related mutations in regulatory proteins lead to continuous division [49]. Disruption of this regulatory mechanism leads to uncontrolled cell proliferation and tumor growth. In particular, overexpression of cyclin D1 and CDK4/6 is common in cellular transformation toward cancer and has been linked to the dysregulation of oncogenes. In contrast, tumor suppressors, such as Rb and p53, which normally halt the cell cycle under unfavorable conditions, are frequently inactivated [50], and CDK inhibitors, such as p21 and p27, which act as natural breaks in the cell cycle, are often downregulated. These disruptions make the G1 phase a crucial target for cancer treatment [50]. CDK4/6 inhibitors such as palbociclib and ribociclib are widely used to block cell cycle progression in cancers [51]. Efforts to restore tumor suppressor function or enhance CDK inhibitor activity have also been made [51,52]. CREPT plays a crucial role in the G1 to S transition as it not only upregulates cyclin D expression but is also involved in the upregulation of other oncogenes responsible for facilitating the cell cycle [9,53]. For instance, in cervical cancer, CREPT expression is significantly upregulated in cancer tissues and is positively correlated with cyclin D1 and transcriptional factor 4 (TCF4) expression. This revealed the role of CREPT in cell cycle progression and tumor growth by facilitating G1/S transition [54]. The same mechanism was observed in salivary gland adenoid cystic carcinoma (SACC), where elevated CREPT expression upregulates cyclin D1, c-MYC, and CDK4 levels and enhances cell cycle progression. However, the downregulation of CREPT reversed these processes, suggesting its critical role in the cell cycle and tumor progression [55]. The impact of CREPT is consistent across various cancer types. In squamous cell carcinoma (OSCC), elevated CREPT expression was associated with cyclin D1 and c-MYC expression and correlated with pronounced stromal interactions and aggressive tumor behavior, highlighting the crucial role of CREPT in promoting aggressive phenotypes [56]. Its elevated expression facilitates the transition from G1 to S phase by upregulating cyclin D3, CDK4, and CDK6 expression in lung and colorectal cancers [27,28]. In gastric cancer, the regulatory role of CREPT is mediated through the p53–p21 axis. CREPT is markedly overexpressed in gastric tumor tissues, and its knockdown induces significant growth inhibition both in vitro and in vivo. Mechanistically, silencing CREPT results in the accumulation of reactive oxygen species (ROS), which activate the tumor suppressor protein p53. This activation leads to an upregulation of the cyclin-dependent kinase inhibitor p21, thereby enforcing G0/G1 cell cycle arrest. Concurrently, p53 activation initiates apoptotic signaling by increasing cleaved caspase-3 and PARP expression levels. Notably, pharmacological scavenging of ROS or genetic suppression of p53 effectively rescues cell viability and prevents apoptosis in CREPT-knockdown gastric cancer cells, confirming the ROS–p53–p21 axis as a key downstream pathway regulated by CREPT (Figure 3). However, riboflavin-depleted conditions induce CREPT expression and suppress p21 and p27 expression, which results in the upregulation of cyclin D1 and CDK4, suggesting that the transcriptional regulation of these genes is associated with metabolic mechanisms that result in cell cycle progression [31]. Moreover, in mouse embryogenesis and development, CREPT plays a critical role and is associated with the upregulation of cyclin D1 and ki67 expression in tissues, while the downregulation of CREPT results in G1 phase arrest by suppressing cyclin D1 and CDK4/6 expression levels. In contrast, CREPT overexpression reverses this phenomenon (Figure 3) [9]. Overarching findings suggest that CREPT mainly promotes the G1 phase through cyclin D1/CDK4/6 in diverse malignancies. The CREPT regulatory network and an understanding of its interaction with cyclins offer insights into its potential for further investigation and possible therapeutic interventions to disrupt the cell cycle machinery in cancer. The overarching theme across these studies is CREPT’s universal role in promoting G1 phase progression through cyclin D1/CDK4/6 upregulation in various cancers. This pattern suggests that CREPT is a converging point in cell cycle regulation and contributes to tumorigenesis across different malignancies. Understanding CREPT regulatory networks and their interactions with cyclins, miRNAs, and metabolic pathways offers valuable insights into potential therapeutic interventions targeting cell cycle dysregulation in cancer.

The D- CDK4/6 regulates the G1-S phase transition, which is often dysregulated in cancer. Therefore, tumor cells mainly depend on the G2-M checkpoint to hinder cell cycle progression and DNA repair. G2-M phase inhibition has become an effective strategy for targeted cancer therapy [57]. In gastric cancer, higher expression promotes cell proliferation by regulating Cyclin B1 expression via its interaction with Aurora B kinase. The study further revealed that CREPT directly interacts with Cyclin B1 in the promoter region, facilitating its transcription and transition from the G2 phase to the M phase of the cell cycle. Aurora B further amplified this process and strengthened its ability to upregulate Cyclin B1. The overexpression of CREPT increased Cyclin B1 expression and enhanced cell division, while silencing the expression of CREPT led to reduced Cyclin B1 mRNA expression. This suggests that CREPT regulates Cyclin B1 at the transcriptional level, delays the transition to G2/M, and ultimately hinders cell growth in cancer cells [22]. This study further analyzed the phosphorylation status of CREPT in eukaryotic and prokaryotic cells. This modification process is essential for enhancing Cyclin B1 expression and promoting the G2/M transition process during tumor growth. These findings were confirmed through dephosphorylation experiments using λ protein phosphatase (λ-PPase), which revealed that CREPT and Aurora B directly interact with each other and phosphorylate it at the S145 site. Thus, increased CREPT enhances Cyclin B1 transcription, ultimately upregulating cyclin-dependent kinase 1 (CDK1) phosphorylation, resulting in rapid cell growth and proliferation [22]. CREPT targeting other therapeutics that disrupt the G2-M phase or other cell cycle checkpoints may significantly improve the treatment efficacy in cancer.

**Table 1:** Key studies of the oncogenic role of CREPT across various human cancers.

Cancer Type	Study Type	Molecular Pathway	Primary Findings	Ref.
Breast cancer	In vitro and in vivo	catenin/TCF4/cyclin D1, miR-138, miR-449b-5p, Wnt/β-catenin	CREPT promotes proliferation/invasion, regulated by miR-138, miR-449b-5p suppresses CREPT, inhibits Wnt signaling, growth/invasion	[11,17]
Tumorigenesis	In vitro and in vivo	STAT3, p300, histone acetylation	CREPT enhances STAT3 transcriptional activity via p-STAT3/p300 complex, promoting tumorigenesis	[58]
Gastric cancer	In vitro and in vivo	ROS/p53, cell cycle, apoptosis, Aurora B, Cyclin B1, G2/M transition	CREPT knockdown induces cell cycle arrest, apoptosis via ROS/p53, inhibits migration, interacts with Aurora B, regulates Cyclin B1, accelerates G2/M transition	[22,31]
Tumorigenesis of multiple cancers	In vitro and in vivo	Wnt/catenin/TCF4, Cyclin D1, RNAPII, chromatin looping, HDAC1, TCF4, Wnt	CREPT acts as co-activator for catenin-TCF4, enhances Wnt signaling, enhances cyclin D1 transcription, competes with HDAC1 for oncogene promoters, promotes oncogene expression	[6,20,59]
Colorectal cancer	In vitro and in vivo	Wnt/β-catenin, p300, histone acetylation, Cyclin D3, CDK4/6, miR-383, Wnt/β-catenin, CCND1	CREPT amplifies Wnt signaling via p300, promotes proliferation/metastasis and cell cycle	[27,30,45]
Glioma	In vitro	miR-596, Wnt/beta-catenin	CREPT promotes proliferation/invasion, regulated by miR-596	[16]
Renal cell carcinoma	In vitro, clinical	Cyclin D1, c-myc, RNAPII, Wnt	CREPT overexpression linked to poor prognosis promotes proliferation/cell cycle	[29]
Hepatocellular carcinoma	In vitro and in vivo	miR-300 suppresses CREPT/Wnt/β-catenin signaling	CREPT expression promotes cell proliferation and can be targeted by miR-300 in HCC	[7]
Diffuse large B-cell lymphoma	In vitro and in vivo and bioinformatics	NF-kB signaling	CREPT promotes proliferation, inhibits apoptosis via NF-kB	[60]
Non-small cell lung cancer	In vitro and in vivo	Cyclin D1, RNAPII	CREPT overexpression promotes proliferation, migration, poor prognosis	[10,28]
Pancreatic cancer	In vitro, in vivo, and PROTAC	RNAPII, cyclin D1, Wnt, PROTAC	CREPT degradation inhibits proliferation, validates as therapeutic target	[61]

## 5. CREPT’s Role in Cell Renewal and Tissue Repair

CREPT is a multifunctional protein that plays a significant role in tissue repair, cell renewal, and tumorigenesis. Elevated expression in tumors facilitates cell progression, survival, and migration. The mechanism involved in higher CREPT expression increases NF-kB/Nrf2 signaling activation, promoting hepatocyte survival and tissue repair during liver injury [23]. In addition to its role in tumorigenesis, it plays a critical role in preserving genomic stability by reducing persistent R-loops, promoting DNA repair, and ensuring rapid cell division and differentiation. CREPT is pivotal for the proliferation and differentiation of Lgr5^+^ intestinal stem cells (ISCs), which are important for epithelial cell repair and homeostasis. The ablation of CREPT in Lrg+ cells led to a reduction in the expression of lineage hallmark genes, such as Neurog3, Pdx1, and Nkx2-2, diminishing the regeneration capability of the intestinal epithelium. CREPT silencing reduced the migration of EdU-labeled cells in addition to the crypt–villus axis, further suggesting a key role in maintaining epithelial turnover. Furthermore, CREPT inhibition downregulates migration-related genes such as Snai3 and Vim, while increasing Lama5 and collagen, which are epithelium-associated genes, confirming its pivotal role in epithelial repair [24]. Additionally, X-ray-irradiated mice exhibited the upregulation of various genes downstream of the Wnt signaling pathway, including CD44, Ascl2, EphB3, and Sox9. However, in CREPT-depleted mice, their expression was impaired, suggesting that CREPT plays an essential role in the activation of the Wnt signaling pathway, contributing to intestinal epithelial regeneration [24]. A new study published recently by Lan et al. (2025) further explored the role of CREPT and identified it as a crucial regulator of revival stem cell (revSC) differentiation during intestinal regeneration using Villin-Cre and inducible Villin-CreERT2-mediated CREPT knockout mouse models, which demonstrated that a loss of CREPT in intestinal epithelial cells leads to regeneration failure following irradiation-induced injury. Notably, CREPT-deficient mice exhibited a significant accumulation of Clu^+^ revSCs but a marked reduction in differentiated enterocytes (ECs) and goblet cells (GCs), suggesting a block in lineage commitment rather than revSC induction. The study further confirmed through single-cell RNA sequencing that CREPT deletion disrupted the transition of revSCs from a naïve state toward absorptive and secretory lineages, as evidenced by altered pseudotime trajectories, reduced CytoTRACE stemness scores, and decreased expression of lineage-specific markers (e.g., Apoa1, Fabp2 for ECs and Muc2, and Tff3 for GCs). Lineage tracing of Clu^+^ revSC progeny confirmed a diminished regenerative output in CREPT knockout mice, with smaller mCherry^+^ clones and fewer Ace2^+^ or Muc2^+^ differentiated cells. Mechanistically, CREPT deletion resulted in a nuclear accumulation of YAP and the upregulation of its downstream targets Areg and Msln, indicating aberrant YAP signaling during the later phase of regeneration. Collectively, these findings establish CREPT as an essential transcriptional regulator that enables revSCs to properly differentiate and contribute to intestinal tissue repair, expanding its known role in stem cell maintenance to include revSC lineage progression under regenerative stress [62].

CREPT influences cell migration and actin cytoskeleton organization by stimulating RhoA and increasing mDia1 expression. This constitutes the CREPT-RhoA-mDia1 signaling pathway, which is responsible for the regulation of focal adhesion and actin filaments and is crucial for cell migration and regeneration. Inhibition of RhoA stimulation diminished actin filament formation, inhibited migration, and compromised the tissue repair mechanism [13]. Together, CREPT functions as a crucial regulator of cell proliferation and differentiation and is involved in the tissue-healing process. These studies have highlighted the importance of CREPT in epithelial tissue repair. Its involvement in RhoA signaling demonstrates a broader function in the cytoskeletal as well as tissue regeneration processes, which makes CREPT a therapeutic target for facilitating tissue repair and regeneration.

## 6. CREPT in Apoptosis Regulation

Apoptosis is referred to as programmed cell death and is an important process through which the body eliminates damaged or unwanted cells to maintain tissue homeostasis [63]. It is one of the hallmarks of cancer, in which the regulation of apoptotic processes is frequently impaired, permitting cells to escape death and grow uncontrollably [64]. The key regulators of apoptosis are pro-apoptotic proteins. In addition to Bak and Bax, B-cell lymphoma 2 (Bcl-2) and B-cell lymphoma 2- extra-large (Bcl-XL) function as anti-apoptotic proteins, and caspases and death receptors regulate this process [65]. An imbalance between these proteins and gene mutations is a hallmark of cancer. For example, Bcl-2 overexpression and p53 dysregulation trigger a balance in cell survival [66]. They may also evade death-receptor-mediated apoptosis by reducing receptor expression or blocking signaling. These disruptions not only drive tumor growth but also lead to resistance to therapies such as chemotherapy and radiation, which rely on apoptosis to kill cancer cells [67]. In diffuse large cell lymphoma, elevated CREPT expression increases the expression of NF-kB, resulting in the upregulation of anti-apoptotic genes such as Bcl-2 and Bcl-xl [60]. CREPT overexpression is also associated with the inhibition of pro-apoptotic caspase-3 and Bax expression. However, the apoptotic effect was elevated upon TNF-α stimulation in CREPT-overexpressing cells [60]. CREPT downregulation reversed this process by reducing Bcl-2 and cIAP1 expression, upregulating caspase 3 and Bax expression, and inhibiting cell survival and tumor progression in lymphoma cells through the NF-kB signaling pathway. Similarly, in gastric cancer, CREPT regulates apoptosis through the ROS-mediated p53 signaling pathway. CREPT knockdown reversed the epithelial–mesenchymal transition (EMT) by downregulating E-cadherin and decreasing N-cadherin, vimentin, and MMP1 expression levels. Apoptosis is associated with increased ROS generation and the upregulation of poly (ADP-ribose) polymerase 1 (PARP), caspase-3, and P53. N-Acetyl Cysteine (NAC) reversed the apoptotic process, which further confirmed that CREPT downregulation resulted in oxidative stress that stimulates the apoptotic process [31]. Furthermore, elevated CREPT levels sensitized colorectal cancer cells to 5-FU treatment and significantly increased the apoptotic cells [8,31]. Additionally, it activates the cyclin-dependent kinase inhibitor p21 and decreases the expression of the anti-apoptotic gene Bcl-2, which is an important gene in apoptotic regulation [31]. Wang et al. showed that exposure of the liver tissue to CCL4 induced apoptosis and necrosis in CREPT knockdown mice [23]. It is well established that CREPT regulates the transitional factor NRF2 [23], which is involved in the inhibition of apoptosis by regulating key apoptotic genes such as Bcl-2, Bcl-XL, and cytochrome-C from the mitochondria and reducing caspases. This mechanism is also involved in establishing resistance to cancer therapeutics such as cisplatin. Thus, the upregulation of NRF2 by CREPT explains its role in the inhibition of apoptosis. Targeting the NRF2/CREPT axis could serve as an important therapeutic strategy and has the potential to increase the sensitivity to treatment. In addition, tumors with low CREPT expression may achieve a better prognosis with therapeutic intervention.

## 7. CREPT’s Role in Signaling Pathway

### 7.1. CREPT’s Role in Wnt/β-Catenin and STAT3 Signaling Pathways

**Figure 4:**
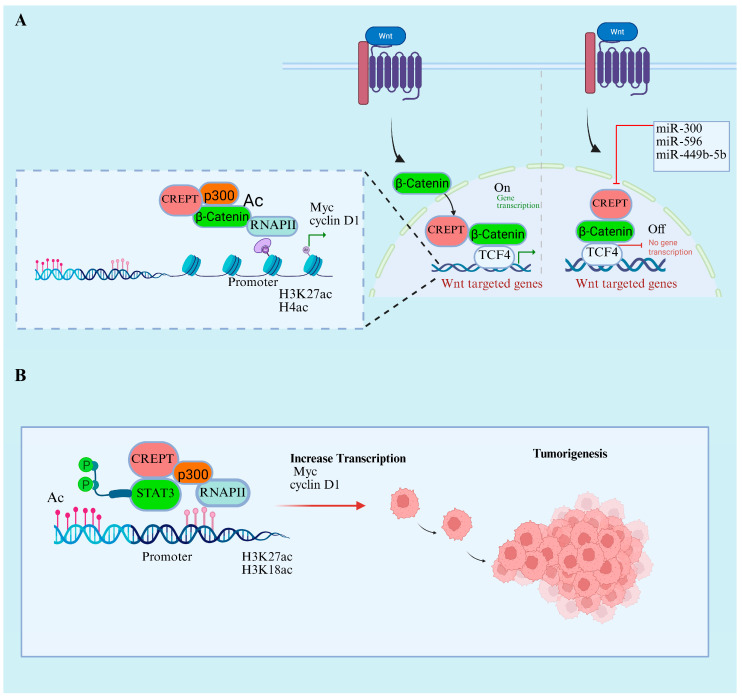
CREPT activity in transcriptional regulation through enhancing acetylation activity. (**A**) CREPT Wnt/β-catenin signaling enhances the target genes transcription through β-catenin nuclear localization. CREPT also enhances p300 acetylation activity of β-catenin at the target promoter and enhances RNAPII activity. (**B**) Similar role of CREPT in enhancing the p300-mediated acetylation STAT3 has also been reported, contributing to enhanced tumorigenesis.

Regulation of the Wnt signaling pathway is required for the normal growth, development, and maintenance of stem cells, and mutation or dysregulation of this pathway can lead to cellular transformation and drive cancer [53]. The oncoprotein CREPT is a critical gene activated downstream of the Wnt pathway in various cancers [7,16,17]. The central mechanism through which CREPT influences Wnt/β-catenin signaling is through its ability to promote the transcriptional activity of β-catenin and TCF4, which are major transcriptional factors in the Wnt signaling pathway, and stabilize their interaction, thereby enhancing the expression of downstream oncogenes, including MYC and cyclin1. miRNAs play a critical role in regulating the CREPT/Wnt/β-catenin signaling pathway. For instance, miR-449b-5b overexpression increases CREPT expression, and the inhibition of miR-449b-5b increases CREPT, β-catenin, and TCF4 expression. This CREPT-mediated transcriptional activation of TCF4 leads to cell invasion and proliferation in breast cancer [59]. Similarly, another miRNA, miR-596, demonstrated an inhibitory effect on CREPT expression, where either CREPT knockdown or the overexpression of miR-596 suppressed Wnt/β-catenin signaling, leading to the restriction of glioma cell proliferation and invasion (Figure 4A). The mechanism of CREPT/Wnt/β-catenin inhibition through miRNAs is consistent across various cancers. Another study revealed that miR-300 has similar effects on CREPT and downstream inhibitory effects through Wnt/β-catenin signaling in hepatocellular carcinoma. This indicates that the CREPT 3′ UTR is pivotal and serves as a target for miRNAs, resulting in the disruption of the Wnt/β-catenin signaling pathway.

CREPT also acts as a co-transcriptional factor that stimulates other key genes such as MYC. The knockdown of CREPT expression impairs MYC transcriptional activity downstream of the Wnt/β-catenin signaling pathway, which leads to the inhibition of cancer cell proliferation [9]. CREPT promotes the p300-induced acetylation of β-catenin and facilitates its transcriptional activity and nuclear localization. In addition to the collective role of p300, Zhai et al. (2021) found that CREPT also engages with phosphorylated STAT3 (p-STAT3) and aids in the recruitment of p300 to the promoters of STAT3-targeted genes. Consequently, CREPT and STAT3 collaboratively enhance the p300-mediated acetylation of histone 3 H3K27 and H3K18ac, thereby increasing the recruitment of RNAPII. The reduction in p300 eliminated the enhancement of STAT3 transcriptional activity by CREPT (Figure 4B) [58]. Additionally, elevated CREPT expression hinders the binding of histone deacetylase 1 (HDAC1) to the promoter of oncogenes, which induces acetylated H3 levels and facilitates the transcription of BMP4, CLDN1, PPARD, and VEGFA. Interestingly, the same study found that elevated HDAC1 expression during CREPT did not influence the transcription of p21 and p27 tumor suppressor genes [20].

The canonical Wnt/β-catenin signaling system governs stem cell self-renewal, cellular proliferation, and cell fate determination within the intestinal crypt microenvironment. The β-catenin transcriptional co-activator regulates the Wnt response; hence, its distribution and levels within a cell are meticulously controlled. CREPT regulates the nuclear retention of β-catenin in crypt cells during intestinal regeneration. This was validated through an immunohistochemical analysis, which revealed nuclear β-catenin in wild-type and CREPT-knockout mice. CREPT deletion also leads to the dysregulation of Wnt signaling and its downstream genes, such as Ascl2, EphB3, and Sox9, and disrupts intestinal epithelial regeneration after X-ray irradiation [24].

### 7.2. CREPT’s Role in NF-kB Signaling Pathway and Tumor Microenvironment

**Figure 5:**
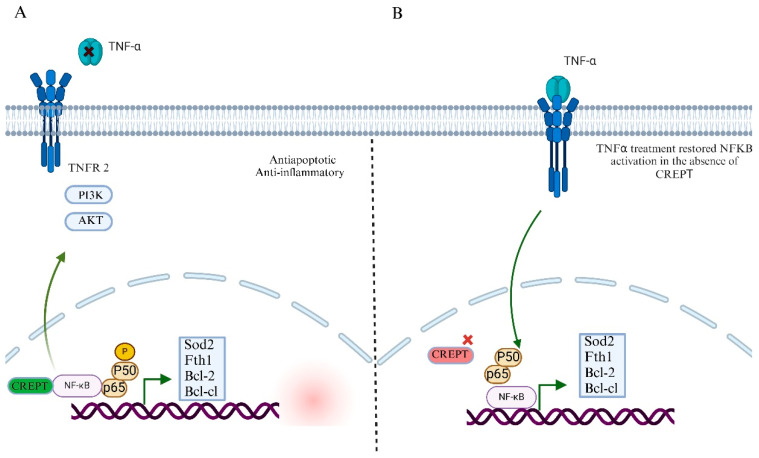
CREPT activates NF-kB/TNFR2 signaling pathway. (**A**) CREPT activates anti-apoptotic and anti-inflammatory effects through stimulating NF-kB signaling pathway. (**B**) CREPT knockdown leads to the downregulation of NF-kB, while TNF-α treatment reverses this process and reactivates NF-kB signaling pathway. This shows that CREPT derives its anti-inflammatory and anti-apoptotic mechanisms through NF-kB signaling.

Nuclear Factor Kapa-B (NF- kB) signaling plays a crucial role in regulating inflammation, immune responses, and cell survival [68]. In cancer, this pathway is often abnormally activated, promoting tumor growth, survival, and resistance to therapy [69]. Under normal conditions, NF-kB remains inactive in the cytoplasm and binds to inhibitors of KB (IκB) proteins [70]. However, signals such as inflammation, stress, or DNA damage activate this pathway, leading to IkB degradation and the release of NF-kB, which then moves to the nucleus to regulate gene expression [70,71]. In cancer, mutations or chronic activations of upstream regulators, such as the IkB kinase (IKK) complex or receptor signaling, lead to persistent NF-kB activation. This supports the expression of genes involved in cell proliferation, angiogenesis, and immune evasion while suppressing apoptosis [72]. Many cancers exploit NF-kB signaling to thrive in hostile environments and evade immune destruction [73]. Several studies have highlighted the crucial role of CREPT in modulating NF-kB signaling [23,60]. This is an important pathway in response to inflammation, apoptosis, and cancer cell proliferation. Dysregulation of this pathway can lead to the activation of anti-inflammatory and immunosuppressive cytokines in cancer [74]. Consequently, this contributes to the inflammation of the tumor microenvironment, leading to immune suppression, tumor development, and therapeutic resistance. Several studies have shown that CREPT positively regulates NF-kB activity and promotes the transcription of downstream genes that contribute to cell survival and proliferation (Figure 5A) [4,23,75]. In diffuse large cell B-cell lymphoma (DLBCL), CREPT inhibits apoptosis by activating the NF-kB signaling pathway. Similarly, CREPT knockdown in lymphoma SU-DHL-2 cells induced apoptosis, whereas this effect was partially reversed by TNF-α treatment, suggesting an important role in stabilizing NF-kB-driven survival signaling (Figure 5B). Further studies revealed that CREPT increases the expression of anti-apoptotic proteins, such as Bcl-2, Bcl-xL, and cIAP1, by upregulating NF-kB transcriptional activity. In addition, CREPT knockdown reduced NF-kB subunit p50 and p65 phosphorylation, implicating CREPT in maintaining the activation of NF-kB signaling [75].

The involvement of CREPT in the NF-kB signaling pathway is beyond hematological malignancies. CREPT knockdown in hepatocellular carcinoma (HCC) leads to the diminished expression of NF-kB/NRF2 target genes, such as Sod2 and Fth1, contributing to the release of reactive oxygen species (ROS) accumulation and impaired liver function [23]. This indicated that CREPT plays a crucial role in protecting hepatocytes and supporting NF-kB-mediated antioxidant responses during oxidative stress, thereby highlighting its importance in cancer progression. For entrance, it is not critical for normal tissue repair and homeostasis and its downregulation in normal liver tissues; however, in cancer cells, its elevated expression leads to tumor progression.

The NF-kB pathway is also critical in oncogenesis, and it has been recently identified that it not only changes the intracellular signaling that drives cancer progression but also plays a critical role in influencing the tumor microenvironment. As a result, the aberrant expression of these genes often leads to the immunosuppressive alteration of cytokine signaling, which affects cells in the surrounding environment and drives the immune cells to an immunosuppressive phenotype. The external receptors on the cells activate intracellular signaling in response to stimuli that mediate signaling through various pathways. The most critical pathways that play a significant role in the interplay between cells and the microenvironment include the Wnt/β-catenin and NF-kB/TNF signaling pathways [76].

Furthermore, extracellular-regulated kinase (ERK) and tumor necrosis factor (TNF) are the two main signaling pathways involved in intracellular signaling [12], and a recent study demonstrated that CREPT acts as an intrinsic mediator in field cancerization, facilitating the transition of normal epithelial cells toward a malignant phenotype under the influence of the tumor microenvironment, particularly through the action of cancer-derived small extracellular vesicles (CDEs) and the activation of signaling pathways like ERK and TNF. Mechanistically, CREPT downstream effector processes include ERK and p38 phosphorylation, which are activated by CDEs. CDEs increase ERK and p38 phosphorylation simultaneously with elevated CREPT protein expression in non-malignant epithelial cells. However, CDE-treated NMuMG and MCF10A cells exposed to the ERK inhibitor, SCH772984, showed decreased CREPT expression. Inhibitors of p38 and JNK, such as SB203580 and SP600125, did not affect CREPT expression. A similar effect of the ERK inhibitor also decreased CREPT expression in 4T1 cells, demonstrating that CDEs activate ERK, which subsequently increases CREPT expression. Furthermore, CREPT suppression can increase the expression of important proinflammatory cytokines such as CCL5, CXCL10, and IL6 through TNFR2 and TNF signaling pathways [12]. These cytokines play critical roles in the reshaping of the immune microenvironment. This suggests that CREPT expression may play a role in altering the tumor microenvironment that influences adjacent cells.

## 8. CREPT’s Role in Metabolic Regulation

Abnormal metabolic processes are a hallmark of cancer, and it has been established that cells require nutrients for growth and division [77]. The role of CREPT in metabolism cannot be ignored as it is a key player in promoting the cell cycle. Recent studies have shown that CREPT enhances fatty acid uptake and synthesis [25]. At the molecular level, higher CREPT expression is associated with an increase in transcription factors, such as c-Jun and c-Fos, which leads to the activation of sterol regulatory element-binding protein 1 (SREBP1), which is crucial in lipid biosynthesis regulation [25]. Additionally, CREPT has been found to be stabilized by long non-coding RNA nuclear paraspeckle assembly transcript 1 (NEAT1) by recruiting the RNA binding protein heterogeneous nuclear ribonucleoprotein A2/B1 (hnRNPA2B1). This stabilization facilitates CREPT expression and further promotes fatty acid uptake and biosynthesis, resulting in lymph node metastasis and tumor growth in gastric cancer [25]. Another study showed that CREPT functions beyond fatty acid metabolism and has been found to interact with various other proteins involved in DNA metabolism and repair processes. In addition, the interaction of CREPT with topoisomerase 1 and RNA helicases facilitates DNA winding and unwinding during transcription [78]. This suggests that CREPT may have broader implications for genomic stabilization and metabolism. However, the current knowledge regarding the role of CREPT in directly targeting the metabolic pathway influencing cancer progression is limited and requires further investigation.

## 9. Targeting CREPT in Cancer Treatment

**Figure 6:**
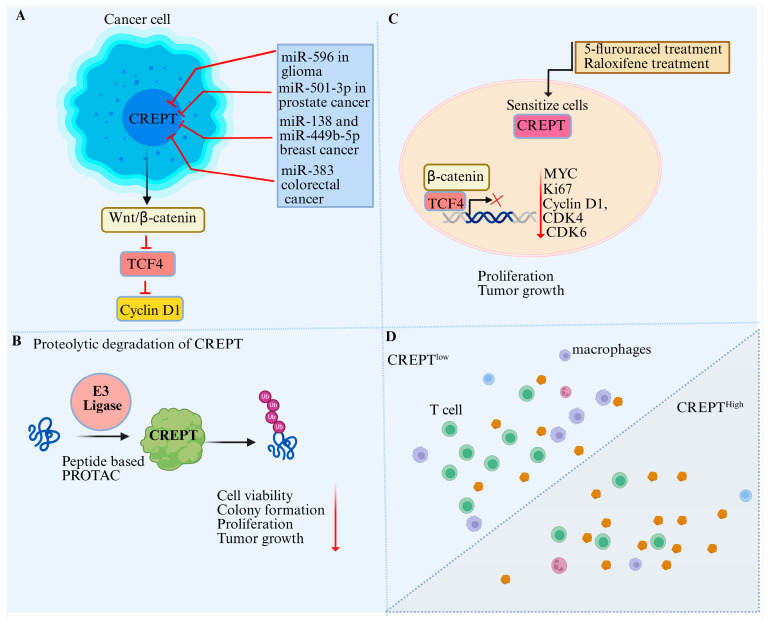
(**A**) CREPT as a target of multiple miRNAs significantly contributes to tumor suppression both in vitro and in vivo. (**B**) CREPT suppression through peptide-based PROTAC has shown useful effects during in vitro and in vivo tumor formation. (**C**) CREPT overexpression has been shown to sensitize cells to 5-FU, while CREPT knockdown sensitizes cells to raloxifene treatments. (**D**) In our proposed model for future studies, we suggest that CREPT expression may influence immune cells and the effect of immune checkpoint blockage based on its role in modulating cytokine expression profile and tumor microenvironment, and should be taken into account in subsequent research.

Despite extensive experimental research over the past two decades demonstrating the role of CREPT in tumor progression, effective strategies to target CREPT expression remain lacking. Several approaches have been explored, including microRNA-mediated regulation [11] or peptide-based PROTACs [61], which have shown promise in inhibiting tumor growth, suggesting their potential as a therapeutic target in cancer treatment. Recent studies have revealed that CREPT, a protein highly expressed in tumors, is regulated by several microRNAs in various cancers. miR-383 targets CREPT in colorectal cancer [10], miR-138 in breast cancer [11], miR-596 in glioma (Figure 6A) [16], and miR-501-3p in prostate cancer [46]. Additionally, miR-449b-5p suppresses breast cancer growth by targeting CREPT [17]. CREPT enhances β-catenin-TCF4 transcriptional activity in response to Wnt signaling [59] and promotes tumor growth by upregulating cyclin D1 expression. Interestingly, miR-454-3p activates Wnt/β-catenin signaling by suppressing multiple pathway antagonists, including RPRD1A/p15RS, in breast cancer. These microRNAs effectively target genes within coding regions, often through repeated sequences [79], highlighting the importance of targeting CREPT via miRNA-mediated regulation in cancer progression.

Targeting CREPT overexpression has been shown to sensitize the cancer cells to various therapeutics. For instance, CREPT overexpression sensitizes colorectal cancer cells to 5-flurouracel (5-FU) treatment. Hence, its overexpression could be used as a potential prognostic biomarker for 5-FU in CRC [8]. CREPT knockdown sensitized endometrial cancer cells to raloxifene treatment (Figure 6C) [21] as CREPT is recognized for enhancing cell cycle progression by transcriptionally activating cyclin D1 and other mitotic regulators, hence promoting G1/S and G2/M transitions. In response to DNA damage, cells generally encounter cell cycle arrest to facilitate repair mechanisms. Nevertheless, CREPT overexpression may evade these checkpoints by sustaining cyclin activity and inhibiting p21 and other CDK inhibitors, so permitting injured cells to persist in proliferation. The reduction in CREPT reduces this proliferative impetus and may hinder the recruitment or efficacy of DNA repair mechanisms. Recent findings demonstrate that CREPT modulates chromatin looping and facilitates RNA polymerase II recycling, which is essential for the production of DNA repair genes [18]. Consequently, its inhibition can interfere with this transcriptional network, undermining DNA damage response pathways. This renders cancer cells more susceptible to chemotherapeutics such as 5-FLU, which causes DNA damage, and raloxifene, which disrupts survival signaling. Therefore, CREPT expression should be taken into consideration in cancer treatment, and its expression profiling may exhibit different responses to treatment and could be used as a biomarker.

Moreover, CREPT suppression can impair DNA damage repair mechanisms, which increases the sensitivity of cancer cells to PARP inhibitors [31]. The interaction of CREPT with Aurora B kinase, cyclin D1, and MYC, which drives the G2/M transition, offers an opportunity to target the CREPT/MYC axis and could significantly improve cancer treatment. These studies indicate that CREPT might need another oncogene, such as MYC, to drive carcinogenesis. Disrupting CREPT expression may impair CREPT/MYC cooperation and could improve immunotherapy in cancer. Therefore CREPT cooperation may also play crucial role in immunotherpy, and stratifying tumors based on CREPT expression levels may provide valuable insights into their immune landscape. Assessing CREPT expression across tumor samples could reveal correlations with variations in T cell infiltration and macrophage polarization, thereby influencing the tumor immune microenvironment. Such classification could also guide therapeutic decisions, particularly in the context of immunotherapy, where high CREPT-expressing tumors might benefit from combined strategies targeting both oncogenic signaling and immune modulation (Figure 6D, unpublished data).

In recent years, significant progress has been made in the development of targeted protein degraders, particularly proteolysis-targeting chimeras (PROTACs). A recent study conducted to degrade CREPT through cell-permeable PROTAC showed a significant inhibition of pancreatic cancer growth (Figure 6B). This is a promising strategy for the inhibition of CREPT-mediated tumorigenesis. We believe that other approaches should also be used in the future to target CREPT expression, such as antisense oligonucleotide. This strategy has shown useful results in targeting other oncogenes such as MYC and has significantly improved the therapeutic efficiency of targeting tumor growth.

Although there are limited studies targeting CREPT in cancer, miRNAs and peptide-based PROTACs degradation have shown significant effects in suppressing tumor growth in preclinical trials. These studies have opened new windows to identify novel therapeutic targets in cancer. We believe that targeting CREPT expressions directly will be more clinically beneficial than the indirect approach for targeting tumor growth. Future efforts will make it possible to identify new agents that directly suppress CREPT expression. As CREPT is highly expressed in cancer cells compared to normal cells, there is a therapeutic margin in which these agents exhibit antitumor efficacy while minimizing toxicity to non-malignant cells.

## 10. Future Direction and Conclusions

CREPT’s role in the regulation of cancer cell intrinsic processes, such as cell proliferation and tumor growth, is evident from various studies. However, there are some limitations, as the majority of the data is derived from in vitro experiments using a narrow range of cancer cell lines, which may not accurately reflect in vivo tumor complexity. Second, the functional consequences of CREPT overexpression or knockdown can vary across cancer types, suggesting possible context-dependent roles that are not yet fully understood. Third, although the role of CREPT has been validated in in vivo models, including conditional knockout mice, patient-derived xenografts are lacking and are needed to validate CREPT’s roles in tumor progression, metastasis, and therapy resistance. Furthermore, a large number of patients should be employed and classified into groups based on the CREPT expression profile. This approach would clarify CREPT’s role in clinical settings, paving the way for therapeutic screening and intervention.

**Figure 7:**
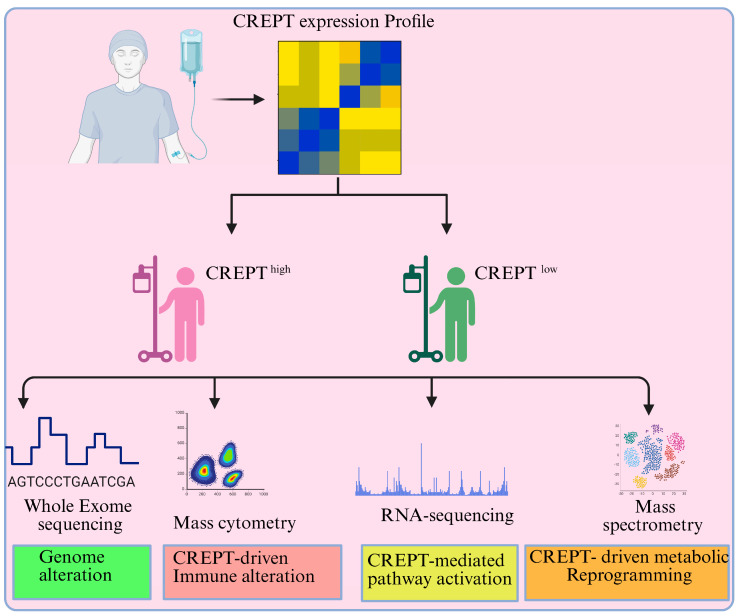
Suggested treatment approaches that use biomarkers to target CREPT-mediated tumor progression. CREPT expression can be evaluated in cancer patients and divided into low and high CREPT groups. Whole-exome sequencing should be performed to investigate genome-wide genetic alterations and compare with CREPT expression profile. It will be useful to investigate how these two groups respond to various therapeutics, such as immunotherapy, to elucidate the role of CREPT in cancer. Tumors with high CREPT expression should be used in preclinical trial studies targeting antisense oligonucleotide, miRNAs, short hairpin RNA, and in combination with immunotherapeutic to study whether targeting CREPT could sensitize the tumor to immune checkpoint inhibitors. These approaches will provide new therapeutic strategies for cancer patients using CREPT as a biomarker and will also clarify the precise process by which CREPT causes cancer.

CREPT is an undruggable oncoprotein that mediates its tumorigenic effects primarily through the Wnt/β-catenin, STAT3, NF-kB, and TNF signaling pathways. Future studies should also be focused on evaluating how CREPT mediates crosstalk between these signaling pathways. These are the key signaling pathways regulating the cell extrinsic phenomena by activating cytokine alteration and playing a critical role in the host immune response and angiogenesis, and they have implications in therapeutic development as well as guiding the therapeutic selection for cancer treatment. To date, CREPT is an undruggable oncoprotein, although we envision that once found, these therapeutics targeting CREPT will be useful in treating CREPT-driven malignancies either alone or in combination with other therapeutics to treat cancer more effectively. Furthermore, the CREPT expression profile of cancer patients may offer alternative therapeutic strategies. Therefore, it is imperative to investigate the patient’s CREPT expression profile and perform a detailed screening to understand how CREPT causes cancer and help determine the therapeutic response to specific therapies. Various screenings, such as DNA and RNA sequencing, cytometry, and spectroscopy analysis, provide directions for selecting therapeutic strategies for patients with CREPT-driven tumor progression (Figure 7). Given CREPT’s pivotal role in cell cycle regulation and its influence on cytokine profiles, we propose that its involvement in remodeling the immune microenvironment warrants greater attention. Future research should explore the impact of CREPT on immune cell populations, particularly macrophages and T cells. Targeting CREPT may enhance the efficacy of immunotherapeutic strategies, including checkpoint inhibitor treatments.

## Data Availability

No new data were generated or analyzed in this study. All information discussed is based on previously published literature, which is cited accordingly.
